# How to Use Multimodality Imaging in Cardio-Oncology

**DOI:** 10.3390/jcdd13010027

**Published:** 2026-01-01

**Authors:** Anca Doina Mateescu, Raluca Ileana Mincu, Ruxandra Oana Jurcut

**Affiliations:** 1 Department of Cardiology, “Prof. Dr. C.C. Iliescu” Emergency Institute for Cardiovascular Diseases, 258 Fundeni Road, 022328 Bucharest, Romania; ruxandra.jurcut@umfcd.ro; 2 Faculty of General Medicine, Carol Davila University of Medicine and Pharmacy, 8 Eroii Sanitari Boulevard, 050474 Bucharest, Romania; 3 Department of Cardiology and Vascular Medicine, West German Heart and Vascular Center Essen, University Hospital Essen, 45147 Essen, Germany; ralumincu829@gmail.com

**Keywords:** cardio-oncology, cancer therapy-related cardiovascular toxicity, cardiovascular imaging, multimodality

## Abstract

Recent advances in oncology have contributed to a steady rise in cancer survivorship. However, many cancer therapies are associated with cardiovascular adverse events, leading to increased rates of cardiovascular morbidity and mortality. As a result, cardio-oncology has emerged as a rapidly advancing discipline that relies on multidisciplinary collaboration. Cardiovascular multimodality imaging (CVMI) is an essential diagnostic and surveillance tool for cardiovascular toxicity, along with clinical evaluation and biomarkers. CVMI plays a central role in diagnosing cancer therapy-related cardiac dysfunction (CTRCD) and myocarditis, while also supporting the assessment of vascular toxicity and arrhythmias. It is essential for baseline cardiac evaluation and continuous monitoring throughout and following cancer therapy. CVMI enables early detection of cardiovascular toxicity, facilitating prompt initiation of cardioprotective therapy and allowing cancer therapy to proceed without compromising safety. Echocardiography is the primary imaging modality for screening, diagnosing, and monitoring CTRCD. Moreover, it is the first-line imaging test for cardiac structural and functional assessment in patients who develop immune checkpoint inhibitor (ICI)-related myocarditis. Advanced imaging techniques, such as cardiac magnetic resonance (CMR), nuclear imaging, and cardiac computed tomography, may help determine the cause and severity of left ventricular dysfunction, as well as assess cardiac masses and vascular toxicity. Not least, CMR is the gold standard imaging modality to diagnose myocarditis. This article is a narrative review that focuses on the various modalities of CVMI and their applications in cardio-oncology. Since the issue addressed is very extensive, this review was designed to be concise.

## 1. Introduction

Cancer and cardiovascular disease are the leading global causes of mortality, imposing substantial economic and societal burdens [1,2]. While recent advances in cancer treatments, such as targeted molecular therapy, immunotherapy, and precision radiotherapy, have led to an increase in the number of cancer survivors, it is well established that many cancer therapies are associated with an increased risk of cardiovascular adverse events [1]. These can range from CTRCD to vascular toxicity, myocardial disease, valvular heart disease, and rhythm disorders [1]. Cancer therapy-related cardiovascular toxicity (CTR-CVT) leads to increased rates of both cardiovascular and oncological mortality, especially when it affects a patient’s ability to complete effective treatments. Consequently, cardio-oncology is a rapidly evolving field that involves multidisciplinary teams. CTR-CVT is a dynamic condition and can occur during cancer treatment or after its completion, from the first year to many years later, warranting close follow-up. Preventing cardiovascular disease is crucial for cancer patients and requires a personalized strategy based on cardiotoxicity risk stratification. This is the key to determining a surveillance strategy that ensures patients undergo optimal cancer therapy while minimizing the risk of CTR-CVT. Assessing the cardiovascular risk can be challenging, so it is essential for clinicians to take a systematic approach without delaying cancer treatment.

CVMI represents, along with clinical evaluation and cardiac biomarkers, an essential diagnostic and surveillance tool for oncological patients at risk of cardiovascular disease. It plays a crucial role in evaluating the severity of pre-existing heart conditions before initiating cancer treatment and establishes a baseline for tracking changes during therapy and long-term follow-up. CVMI can facilitate the timely detection of cardiac dysfunction, enabling the early start of cardioprotective medications. In many cases, this allows for the safe continuation of cancer therapy. Echocardiography is the primary imaging method used for screening, diagnosing, and monitoring cancer therapy-related cardiac dysfunction (CTRCD). Moreover, is the first imaging modality when assesing patients who develop ICI-related myocarditis. Advanced imaging techniques, such as cardiac magnetic resonance (CMR), nuclear imaging, and cardiac computed tomography (CCT), may be helpful for determining the cause and severity of left ventricular (LV) dysfunction, as well as for assessing cardiac masses and vascular toxicity. Not least, CMR is the gold standard imaging modality to diagnose myocarditis. This article is a narrative review that focuses on the various modalities of CVMI and their applications in cardio-oncology. Since the issue addressed is very extensive, the review was designed to be concise. However, one significant limitation of the present review is its narrative character, which makes it vulnerable to subjectivity and bias given the researcher’s involvement.

## 2. Cardiotoxicity—Types and Definitions

Several types of cardiovascular toxicities should be recognized/identified, with CVMI playing a key role in their detection (Table 1). According to current guidelines, we will use the term “CTRCD” to describe cardiac injury, cardiomyopathy, and heart failure (HF) [1]. The CTRCD definition, with its two subforms—symptomatic and asymptomatic—is detailed in Table 2 [1]. The most frequent clinical manifestation of cardiotoxicity induced by chemotherapy is HF [3]. However, with the advancement of immuno-oncology therapies, myocarditis has become an increasingly recognized cardiotoxicity that may or may not be linked to a decrease in LVEF. Additionally, coronary artery disease (CAD) has been associated with antimetabolites, particularly 5-fluorouracil, as well as newer molecules like VEGF inhibitors or tyrosine kinase inhibitors (TKI) [1,4]. Hypertension is also a relatively common side effect of various antineoplastic therapies, particularly noted with VEGF inhibitors [1]. The definitions of other CTR-CVT, including pericardial and valvular heart diseases, remain consistent with those used for the general cardiology population, excepting CTRCD, where the definitions substantially differ from the classical definition of HF [5]. CVMI can provide complementary information useful for the assessment of selected patients with cancer.

## 3. Role of CVMI in Baseline Risk Stratification and Surveillance in Patients Undergoing Potentially Cardiotoxic Cancer Treatment

### 3.1. Echocardiography

#### 3.1.1. Baseline Assessment

Transthoracic echocardiography (TTE) is a non-invasive diagnostic test and is the preferred imaging technique for baseline risk stratification in cancer patients due to its accessibility, low cost and lack of radiation. All cancer patients who have pre-existing cardiovascular disease or are at risk for CTRCD should undergo a baseline TTE [1]. Echocardiography provides information regarding cardiac structure and function which may influence the therapeutic decision. It allows quantitative assessment of LV and right ventricular (RV) size and systolic function, regional wall motion abnormalities, LV diastolic function, valvular heart disease, pulmonary arterial pressure, and pericardial disease [1].

Table 3, Table 4, Table 5 and Table 6 include recommendations for transthoracic echocardiography at baseline and follow-up for classical chemotherapies (e.g., anthracyclines), targeted therapy (HER2 and VEGF), and ICI.

##### Left Ventricular Systolic Function

**Figure 1:**
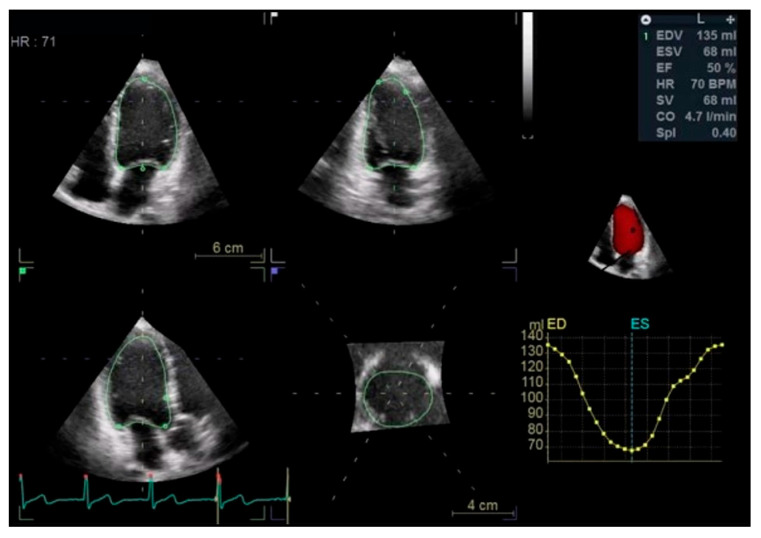
The assessment of left ventricular ejection fraction by 3D transthoracic echocardiography.

LV EF is the most commonly used method for assessing LV systolic function [6]. Patients with a baseline LV EF of less than 50% or in the low normal range (LVEF of 50–54%) are at the highest risk for cardiotoxicity from anticancer therapies. However, standard 2D TTE for LV EF assessment relies on geometric assumptions, which limit its accuracy and reproducibility. In contrast, three-dimensional echocardiography (3DE) overcomes these limitations [1,7,8]. Therefore, 3DE is the preferred echocardiographic modality for evaluating LV EF and cardiac volumes. (Figure 1) The modified 2D Simpson’s biplane method is recommended when 3DE is not feasible [1].

The accuracy of the LV EF volume measurements by TTE may be compromised by a poor quality of the acoustic window in obese patients, after radiation therapy, or recent surgery (e.g., mastectomy), thus limiting the adequate visualization of the endocardial border. In these cases, contrast echocardiography should be considered for a more accurate assessment of LV EF [1,9]. LV opacification is recommended when the visualization of two or more LV segments is poor [10].

While LV EF carries important prognostic information and is the basis for many therapeutic decisions, it is not a direct measure of contractility and does not describe intrinsic myocardial function. Instead, LV EF reflects only the volume changes during the cardiac cycle and is influenced by factors such as volume, preload, afterload, heart rate, and valvular function [5]. Previous studies in the field of cardio-oncology have demonstrated that there is a significant need for an integrated approach that incorporates complementary imaging techniques to provide a comprehensive assessment of LV systolic function. Thus, combining LV EF with more sensitive parameters of LV dysfunction has become essential.

Two-dimensional speckle-tracking (STE) analyzes the myocardial deformation on 2D images by tracking natural acoustic reflections and interference patterns called “speckles.” The software can provide the deformation between speckles within a predefined region of interest, obtaining a value defined as “strain.” Compared with LV EF, GLS is a more sensitive and reproducible measure of LV systolic function and has emerged as an early marker of cardiotoxicity [11,12,13]. Therefore, when available, current guidelines recommend baseline LV GLS measurement for all patients undergoing TTE before cancer treatment [1].

If GLS cannot be assessed, alternative markers of longitudinal function should be considered. Tissue Doppler imaging (TDI) may detect impairment of longitudinal function as an early marker of LV dysfunction that precedes the drop in LV EF [14]. TDI allows the preferential sampling of myocardial motion and is most often recorded using pulsed-wave (PW) or color Doppler. PW-Doppler TDI technology measures the velocity of individual myocardial segments. Strain-TDI is then calculated as the change in myocardial tissue length over time, derived from TDI velocity data. However, LV deformation analysis using TDI is angle dependent, it cannot be performed for all LV segments, and suffers from noise, translational movements, aliasing and reverberation. For these reasons, TDI analysis has been replaced by STE, which overcomes these limitations, allowing a multidirectional evaluation of myocardial deformation [15].

Systemic arterial blood pressure should be assessed in all patients undergoing baseline TTE, and the results should be documented on the TTE report, as loading conditions can change frequently during chemotherapy. These changes may affect cardiac volumes, LV EF, and GLS measurement [1]. Myocardial work (MW) assessment overcomes the load dependency limitation of GLS, incorporating blood pressure measurements. MW offers a more complete picture of the LV function by quantifying both constructive as well as wasted work [16]. Although MW indices have diagnostic and prognostic value in various cardiovascular conditions, their role in cardio-oncology is still unclear [17,18]. To date, few studies have assessed the potential role of MW analysis in patient risk stratification and early detection of CTRCD [19,20]. Moya et al. demonstrated that baseline quantification of MW indices allowed for the identification of cancer patients at higher risk of developing CTRCD [19]. In contrast to the work of Calvillo-Argüelles et al., Moya et al. demonstrated that not a higher wasted work, but rather lower constructive work was responsible for the depressed myocardial work efficiency at baseline in patients with moderated CTRCD during follow-up [19,20]. These findings may suggest a distinct phenotype with a higher susceptibility to developing CTRCD, characterised by a less efficient LV performance due to lower contractile capacity. However, MW is less available in clinical practice.

##### Left Ventricular Diastolic Function

Evaluating LV diastolic function by TTE is crucial for a thorough baseline assessment in patients with suspected or confirmed HF diagnosis. Relevant cardiac structural changes include pathological LV hypertrophy and dilation of the left atrium (LA). On the other hand, functional abnormalities, such as alterations in LV relaxation, compliance, or stiffness, are best assessed by mitral inflow velocities, mitral annular e’ velocities, the E/e’ ratio, and tricuspid regurgitation velocity [21]. In cardio-oncology, baseline LV diastolic function does not predict CTRCD, and evidence supporting this is limited [22,23]. Randomised studies in larger populations, using new parameters, are required to define the role of LV diastolic dysfunction in the early diagnosis of cardiotoxicity induced by antineoplastic therapies.

##### Left Atrium

Atrial fibrillation (AF) is a common adverse event in cancer patients, especially in those receiving Bruton Tyrosine Kinase inhibitors (BTKi). Until recenty, studies have reported an incidence of ibrutinib-related atrial fibrillation (IRAF) up to 6–16%. However, recent real-world data showed a higher incidence compared to clinical trials [24]. Therefore, identifying risk factors for the future development of AF in cancer patients treated with IBTK has gained interest in the last few years. Various clinical conditions predicted future development of IRAF in these patients, such as male gender, older age and the presence of one or more cardiac comorbidities, e.g., prior HF [25]. LA abnormality on electrocardiogram and LA dilation on TTE in terms of antero-posterior diameter (with cut-off value of 32 mm) and area respectively (with cut-off value of 18 cm2), also predicted future development of IRAF in cancer patients receveing IBTK [25,26,27]. Very few studies evaluated the relationship between IRAF and LA function using advanced echocardiography. Arushi et al. showed that peak LA longitudinal strain and peak LA contraction strain remained significantly associated with the development of IRAF [28]. However, future prospective studies are needed to determine whether these findings can identify an increased risk of IRAF in these patients, enhance surveillance, adjust anticoagulation management, and possibly alternate oncologic therapies.

##### Right Ventricular Size and Function

Assessment of right ventricular (RV) function is increasingly important in patients undergoing chemotherapy, radiotherapy, or immunotherapy. RV dysfunction is known to be a parameter of poor prognosis in different cardiovascular diseases [29,30,31]. Compared with the LV, the RV has a significantly smaller mass and thinner walls. Therefore, the RV myocardium reacts more rapidly than the LV to volume and haemodynamic changes. This could explain the RV’s higher susceptibility to the administration of cardiotoxic ICIs compared with the LV [32].

Baseline quantitative assessment of RV size and function is also recommended in cancer patients [33]. Standard RV assessment by TTE routinely includes RV dimensions, RV S’, tricuspid annular plane systolic excursion (TAPSE), and pulmonary artery systolic pressure. In addition to standard parameters, 2D RV strain is recommended, although RV deformation analysis is still being investigated for detecting subclinical RV dysfunction in cancer patients [1].

##### Stress Echocardiography—Functional Myocardial Ischemia Assessment

In all cancer patients with pre-existing CAD baseline TTE is recommended [1]. Moreover, stress echocardiography is helpful to assess for occult obstructive CAD, as such patients may be at increased risk for major CV events.

In asymptomatic cancer patients with high or intermediate pretest probability for CAD, stress echocardiography allows the evaluation of inducible myocardial ischemia, especially in those who are planning to receive antineoplastic drugs associated with vascular toxicity like fluoropyrimidines, VEGF inhibitors, breakpoint cluster region–Abelson oncogene locus, and TKIs [1,34].

In symptomatic cancer patients stress echocardiography is potentially helpful for risk stratification if clinical suspicion of CAD exists. However, ensuring such a work-up should not delay the necessary cancer therapy is essential.

Moreover, stress echocardiography may have a role in identifying LV dysfunction in patients receiving ICI therapies. As evidence, an experimental model conducted by Michel et al. showed that ICI therapy, including the PD1 inhibitor, is associated with significant cardiac adverse effects that affect immune homeostasis, leading to baseline LV dysfunction and inotropic stress with dysregulated metabolism [35]. These data indicate that ICI cardiotoxicity is not limited to myocarditis, and we must consider a more global effect.

The advantages of exercise or pharmacological stress echocardiography are that it requires no radiation and is highly feasible and low-cost.

#### 3.1.2. Surveillance and Identification of CTRCD

##### Left Ventricular Systolic Function

Current definitions of CTRCD rely on LVEF reduction and/or relative changes in LV GLS Table 2 [1]. Thus, LV EF and strain are recommended for the detection of CTRCD during and after cardiotoxic cancer treatment. TTE allows quantitative assessment for serial surveillance. The same imaging modality used for LV EF and GLS baseline evaluation should be ideally used for serial surveillance. The frequency of serial echocardiograms depends on the type and dose of anticancer agent and on symptoms [1]. A relative change in GLS must be compared with the previously reported measurements [36].

Recent advancements in the semi-automated assessment of 3D LV EF in clinical settings have decreased the temporal variability in LV EF measurements. This improvement is evident in both intra- and interobserver variability, as well as in test–retest variability. This enhancement is particularly significant in CRTCD where serial evaluation of LV systolic function is needed. LV volume measurements obtained through real-time 3DE are more reliable and accurate than those obtained by 2DE [6,7,8]. Comparisons between 3DE LV volume determinations and those derived from 2DE and CMR imaging revealed that LV volume is significantly underestimated by 2DE but much less so by 3DE [37]. A study by Mor-Avi et al. demonstrated that the accuracy of 3DE in volumetric assessments is comparable to that of the gold standard CMR [38].

Contrast echocardiography has shown additional value, significantly reducing the interobserver variability of LV volumes and wall motion score index [39]. Specifically, the use of 2D contrast echocardiography resulted in a reduction in the interobserver variability of LV EF from 14.3% to 8% [40]. Conversely, the value of RT-3D contrast echocardiography remains uncertain. Hoffmann et al. reported a reduction in interobserver variability from 14.3% to 7.4% [40], while Thavendiranathan et al. found no significant added value compared to non-contrast RT-3D echocardiography [41].

The assessment of LV systolic function only using LV EF is limited due to its late impairment in various cardiovascular diseases, making it insufficient to detect subclinical LV dysfunction.

To date, there is controversial data regarding the utility of the analysis of LV systolic function using TDI in cardio-oncology. In their study, Fallah-Rad et al. demonstrated a significant reduction in lateral myocardial S velocity within three months of chemotherapy initiation. All these patients later developed LV dysfunction [42]. However, this study had several limitations, the most important being the high incidence of cardiotoxicity in a relatively small and young population. Moreover, Jurcut et al. showed that myocardial deformation parameters obtained with Doppler-based imaging enabled the detection of subtle changes in both longitudinal and radial LV function after six cycles of pegylated liposomal doxorubicin [43]. Therefore, the authors suggested that Doppler-based myocardial deformation imaging should be used for cardiac function monitoring during chemotherapy with anthracyclines. However, the main limitation of this study was the reduced cancer population [43]. On the other hand, other studies failed to prove a significant reduction in myocardial S velocity in chemotherapy-treated cancer patients [44,45].

The 1 year results of the SUCCOUR trial showed that treating various cancers patients who are receiving cardiotoxic therapy with angiotensin-converting enzyme inhibitors and beta-blockers, based on decreased LV GLS, lead to a smaller decline in LV EF than if treated after the LV EF had already decreased [46]. However, during the 3 years follow-up, the differences in the incidence of cardiotoxicity between GLS-guided management and LVEF-guided management disappeared [47]. Therefore the latest clinical guidelines and several other recommendations do not support the isolated finding of altered GLS values as a sufficient parameter to guide therapy.

Despite extensive research on LV GLS concerning chemotherapy and targeted therapies, limited studies focus specifically on patients receiving ICI theraphy. Coskun et al. tried to adress this gap and retrospectively studied 44 patients that received ICI therapy and underwent pre- and post- treatment assessments of LV EF and LV GLS [48]. In their study, LV GLS reduction did not demonstrate a significant role in the early prediction of ICI-related myocarditis or cardiac dysfunction. Further multicenter, large-scale and prospective studies with extended follow-up periods are needed to assess the role of LV GLS in predicting CTRCD and guiding therapy in patients receiving ICI.

**Figure 2:**
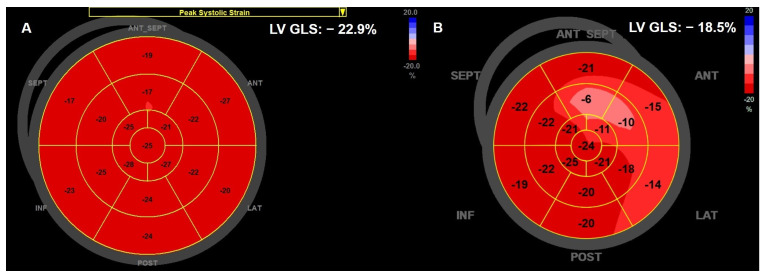
Two-dimensional speckle-tracking LV GLS displayed in the bull’s eye format. (**A**) Normal global and regional LV longitudinal strain (GLS −22.9%) in a 37-year-old female patient with breast cancer and associated cardiovascular risk factors (current smoker, hypercholesterolemia) who received prior treatment with doxorubicin and was referred for cardiological evaluation before initiating trastuzumab therapy. We also note a normal LV EF of 60% at this evaluation. (**B**) After six cycles of trastuzumab therapy, we observed a decline in LV GLS (−18.5%). In the same patient, LV EF was not significantly altered. 2D: two-dimensional; LV: left ventricular; GLS: global longitudinal strain.

2D-STE provides an accurate definition of longitudinal, circumferential, and radial LV deformation. Among all these LV deformation components, GLS is the most sensitive parameter for detecting early LV systolic dysfunction [36,49]. (Figure 2) However, GLS determination using STE has several limitations, and an important one is represented by intervendor variability in normal strain values [33]. Thus, the current recommendation is to use the same vendor for GLS assessment during follow-up [11]. This is important for meaningful comparison and early detection of CTRCD in multiple cancers and treatments.

Global circumferential strain has been reported to identify patients at risk of CTRCD [50]. Quinaglia et al. showed that global circumferential strain (GCS) and global radial strain (GRS) are lower in patients with ICI myocarditis than in those receiving ICI who did not develop myocarditis [51]. Lower GCS and GRS values were observed in patients presenting with both preserved and reduced LVEF. Also, during follow-up, lower GCS and GRS predicted subsequent adverse cardiovascular events after ICI myocarditis diagnosis with greater accuracy than other traditional parameters, such as biomarkers and LVEF, used for risk stratification in ICI myocarditis. However, data are currently insufficient to recommend its routine use.

A future potential echocardiographic parameter is 3D LV strain. 3D-STE is one of the most advanced techniques in the evaluation of myocardial deformation. It includes no through-plane motion of speckles and the ability to track speckles in 3D space, avoiding the errors derived from the use of 2D images. This allows the calculation of circumferential, radial, and longitudinal strain in one measurement. Recent studies reported that 3D-STE LV GLS is slightly less feasible than 2D-STE. However, 3D-STE appeared less time-consuming, and the correlation between values obtained by the two methods was good [52]. Yu et al. demonstrated that childhood cancer survivors evaluated by 3D-STE had significantly reduced GLS and torsion and greater systolic dyssynchrony index compared to healthy controls [53]. Mornos et al. found that 3D-STE GLS was superior to biomarkers and to LVEF in predicting cardiotoxicity development [54]. Although exciting, more studies are needed in this area. Moreover, 3D STE is not widely available in all echo laboratories, thus its use remains experimental.

##### Left Ventricular Diastolic Function

Previous studies have indicated that changes in LV diastolic function occur with anthracycline cancer therapy [55,56,57,58]. However, these studies have primarily focused on anthracyclines alone, had limited follow-up durations, and did not utilize the newer classifications of LV diastolic function grades or examine their associations with subsequent declines in LV systolic function.

The largest prospective study to date evaluating LV diastolic function changes due to breast cancer therapy has revealed a decline in LV diastolic function early on following doxorubicin exposure, which persisted over the long term [22]. The study found that the worsening of LV diastolic function from baseline was linked to a 1.4% reduction in LV EF from baseline and a 2.2-fold increased risk of developing CTRCD [22]. These findings indicate that significant changes in LV diastolic function occur over time with modern breast cancer therapies. Abnormal or worsening LV diastolic function precedes systolic dysfunction and is associated with an elevated risk of CTRCD. A significant clinical implication is the need for aggressive modification of associated cardiovascular risk factors related to LV diastolic dysfunction in the general population. This includes measures such as rigorous blood pressure management, weight loss, and regular exercise, which should be implemented before the onset of worsening LV diastolic function to potentially delay the progression to more severe diastolic dysfunction and the development of LV systolic dysfunction [59].

##### Left Atrium

**Figure 3:**
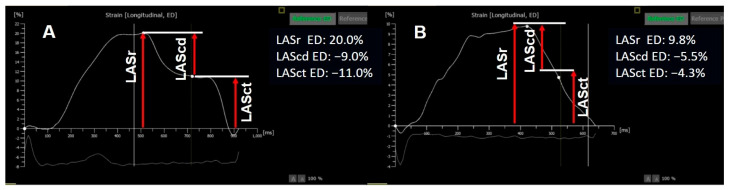
A 65-year-old female patient, with a 12-year diagnosis of chronic lymphocytic leukemia and unfavourable evolution under multiple antineoplastic therapies, some with cardiotoxic potential, with relapse of the disease. She was referred for baseline cardiologic evaluation due to increased risk of cardiotoxicity. Following the assessment, she was started on the second-generation Bruton’s tyrosine kinase inhibitor, acalabrutinib. (**A**) Baseline echocardiographic evaluation showed normal phasic components of left atrial strain but with reduced LASr (20%). (**B**) One month after initiating acalabrutinib, the patient developed paroxysmal atrial fibrillation. Reevaluation of her echocardiogram indicated a decline in all phasic components of left atrial strain, while her left atrial volume remained unchanged. LASr: LA strain reservoir; LAScd: LA strain conduit; LASct: LA strain contractile.

Currently, LA size or function are not included in the definition of CTRCD nor in the risk scores for developing this condition. However, some studies have explored its role in the field of cardio-oncology. For instance, Bergamini et al. found that patients with a dilated LA and normal LV function, as observed in baseline TTE before undergoing adjuvant or neoadjuvant trastuzumab therapy, developed CTRCD during treatment [60]. Furthermore, a study by Park et al. indicated that a decline in peak LA longitudinal strain at the end of chemotherapy predicted CTRCD with better sensitivity and specificity than LV GLS [61]. A combined strategy using clinical variables (smoking and prior cardiovascular disease), the evolution of LV GLS, LA reservoir strain (relative declines in LV GLS [≥6.5%], and LASr [≥7.5%]) and cardiac biomarkers (TnT ≥ 0.019 ng/mL and BNP ≥ 31.1 pg/mL at 3 months) was proposed to divide patients in low, intermediate and high risk categories for further CTRCD development [62]. (Figure 3)

##### Right Ventricle

RV function has not been incorporated into the definition of CTRCD. Emerging evidence suggests that RV dysfunction can occur during cancer treatment, though still limited by small studies [63,64]. RV abnormalities have proven prognostically significant in cancer patients undergoing anthracycline and trastuzumab therapy [63]. Pohl et al. retrospectively examined 30 patients (40% women, age 59 ± 13 years) with advanced melanoma before and 4 weeks after the start of ICI therapy [64]. ICI therapy caused a reduction of RV free wall longitudinal strain even in a short-term follow-up. In contrast, changes in RV function were not visible by conventional RV parameters, including TAPSE and fractional area change. These findings suggest that RV strain analysis should be performed in patients undergoing ICI therapy, as alterations in RV deformation may be early signs of cardiotoxicity. The mechanism of subclinical RV dysfunction in patients receiving anticancer treatment remains unclear. Fluid overload after anticancer therapies may affect the right heart function. However, ICI therapy is associated with a low infusion volume, and conventional echocardiographic RV parameters susceptible to fluid overload were similar between both time points. Prospective studies involving large populations of cancer patients are needed to establish the prognostic role of RV function in this setting and its clinical impact on therapy.

##### Pulmonary Hypertension

TTE is also helpful for screening pulmonary hypertension (PH) in asymptomatic cancer patients who received drugs that potentially induce group 1 PH, such as TKIs, particularly dasatinib and ponatinib. Estimating the incidence of PH related to these agents is challenging due to small study sizes and the absence of screening data from asymptomatic patients.

According to current guidelines, TTE should be considered every 3 months during the first year of treatment for asymptomatic high-risk patients while receiving dasatinib or ponatinib [1]. For those patients who require long-term (>12 months) treatment with these medications, TTE may be considered every 6 to 12 months [1].

Although dasatinib-induced PH is often reversible, it does not typically return to normal baseline pulmonary pressures. This highlights the need to enhance strategies for the early identification of at-risk patients [65].

### 3.2. Cardiac Magnetic Imaging

#### 3.2.1. Baseline Assessment

When available, CMR should be considered for assessing cardiac function when TTE is unavailable or non-diagnostic [1].

CMR is the gold standard for measuring LV volumes and function as its volumes are not based on geometric assumptions and are independent of suboptimal imaging. Therefore, CMR provides the most accurate evaluation of both global and regional myocardial dysfunction [66,67,68].

Moreover, if a specific cardiovascular disease is identified in cancer patients, e.g., hypertrophic cardiomyopathy, CMR should be considered for further risk assessment [1].

**Figure 4:**
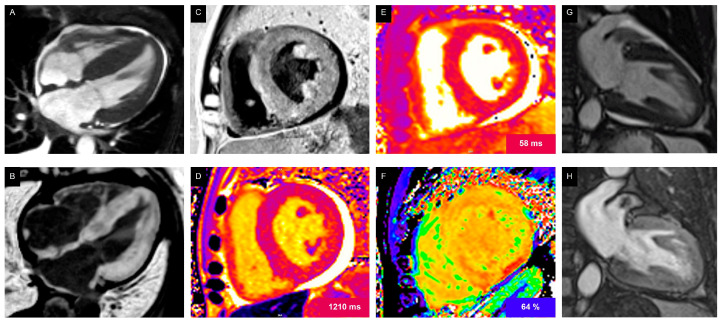
Cardiac magnetic resonance (CMR) imaging sequences from patients with cardiac amyloidosis. (**A**): Cine (bSSFP) four-chamber, systolic frame showing biventricular wall thickening, thickened atrial walls and interatrial septum, as well as pericardial and right-sided pleural effusions. (**B**,**C**): Late gadolinium enhancement (LGE) sequences in four-chamber and short-axis views demonstrating diffuse transmural LGE with involvement of the right ventricular free wall, both atrial walls, and the interatrial septum. Abnormal gadolinium kinetics resulting in a “dark blood” appearance are also visible. (**D**,**F**): Parametric mapping showing elevated native T1 (**D**), T2 (**E**), and extracellular volume fraction (ECV) after contrast administration (**F**). (**G**,**H**): Cine two-chamber view (**G**) depicting a suspected mass, which was confirmed as a left atrial appendage thrombus on the early gadolinium enhancement (EGE) sequence (**H**) in a patient with light-chain (AL) amyloidosis.

CMR is also recommended in patients with suspected cardiac amyloidosis (CA) due to light-chain (AL) amyloidosis. AL amyloidosis is a systemic disease that can occur in conjunction with other haematological disorders like multiple myeloma or Waldenstrom disease, or independently as a light-chain protein-producing disorder. [1] CMR with LGE and parametric imaging has emerged as a non-invasive gold standard for AL-CA diagnosis. The following modifications identified in CMR are supportive of AL-CA: diffuse subendocardial or transmural LGE, elevated native T1 values, abnormal gadolinium kinetics, and extracellular volume ≥ 0.40% [1] (Figure 4).

While not specifically tested in cardio-oncology patients, stress CMR may be helpful to assess for myocardial ischaemia in symptomatic patients if clinical suspicion of CAD exists, especially before initiation of cancer therapies associated with vascular toxicity [69].

#### 3.2.2. Surveillance and Identification of CTR-CVT

##### Myocardial Dysfunction

**Figure 5:**
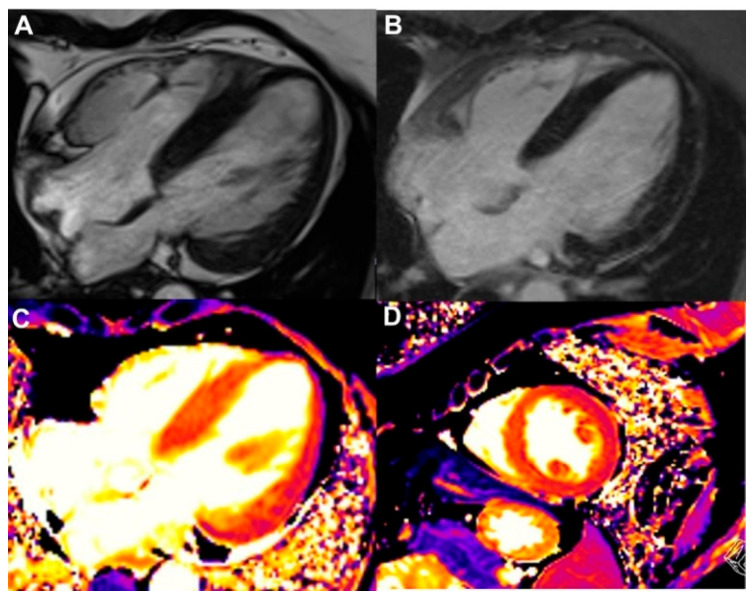
Left ventricular myocardial dysfunction secondary to chemotherapy in a 56-year-old patient treated with trastuzumab. (**A**) Cine (bSSFP) four-chamber view demonstrated mildly reduced left ventricular ejection fraction (LVEF 50%). (**B**) Late gadolinium enhancement shows no evidence of myocardial scar. (**C**,**D**) Native T1 mapping in both four-chamber and short-axis views reveals diffuse myocardial fibrosis, reflected by prolonged relaxation times.

The role of CMR may be limited for the baseline assessment in cardio-oncology. However, its versatility makes it an important imaging modality for identifying CTRCD. The definition of CTRCD is based on an LVEF decline, reiterating the importance of an accurate and reproducible imaging. CMR is superior to 2D TTE in identifying LV dysfunction in patients treated with potentially cardiotoxic medications. (Figure 5) Previous studies demonstrated a decrease in LVEF and LV mass as assessed by CMR in asymptomatic adult survivors of childhood cancer who were treated with anthracyclines, while other imaging techniques failed to detect myocardial alterations [70,71,72].

Moreover, CMR is a valuable tool in certain scenarios, particularly for patients diagnosed with cardiotoxicity through echocardiography and in whom the interruption of treatment could be inadvisable. Additionally, it helps resolve discrepancies in LV EF assessments observed in serial echocardiograms. By confirming LV dysfunction and investigating its underlying causes, CMR plays an essential role in guiding the effective and safe initiation and surveillance of potentially cardiotoxic cancer therapies.

CMR can also measure myocardial strain. Data on CMR myocardial strain correlate with a decline in LVEF. However, its prognostic ability has not been assessed to the extent it has been in echocardiography [73,74,75].

##### Detection of Early and Late Cardiotoxicity

Myocardial toxic effects of chemotherapy ranges from tissue oedema and necrosis to focal replacement and interstitial fibrosis. Use of multiparametric CMR allows assessing this process, combining conventional sequences with T1/T2 mapping imaging. These include the quantification of extracellular volume, as an indirect biomarker of tissue fibrosis and interstitial space expansion [76]. Therefore, CMR is useful to investigate both early and late myocardial dysfunction in chemotherapy treated patients.

Generally, an increase in pre-contrast T1 is associated with myocardial oedema, inflammation, and fibrosis. In contrast, the increase in T2 relaxation time is associated with acute myocardial oedema, a water-sensitive process [77,78]. Cardiotoxic drug exposure has been associated with an increase in both native T1 and T2 relaxation time [79]. As an early acute hallmark of cardiotoxicity, many studies stress the pivotal role of increased T2 relaxation time [80]. A significant signal T2 intensity increase was noted after three days of chemotherapy and predicted LV EF reduction after one year of cardiotoxic treatment [81]. Anthracyclines and trastuzumab treatment in HER-2 positive breast cancer patients is associated with an elevated T2 mapping still at a subclinical stage of cardiotoxicity without any LV EF decrease [82].

Tissue characterization also includes the use of qualitative/semi-quantitative methods based on the administration of gadolinium contrast agents. The composition of the extracellular matrix is altered in myocardial fibrosis. This structural change allows gadolinium to accumulate in areas of replacement fibrosis. On T1 weighted sequences, regions of gadolinium accumulation appear hyperintense and bright, whereas in a healthy myocardium, it appears dark. The data on the utility of LGE in CRT-CD is conflicting. The majority of short-term studies have not reported any LGE [74,83,84]. However, longer-term follow-up studies document LGE incidence of 5–19% [85,86]. The most comprehensive and up-to-date study on LGE regarding anthracyclines, with or without trastuzumab, reveals an incidence of 10% [87]. Notably, nearly all cases examined in this study presented with alternative causes for LGE, which compellingly challenges the efficacy of LGE as a marker for CRT-CD associated with anthracyclines and/or trastuzumab [87].

##### Myocarditis

**Figure 6:**
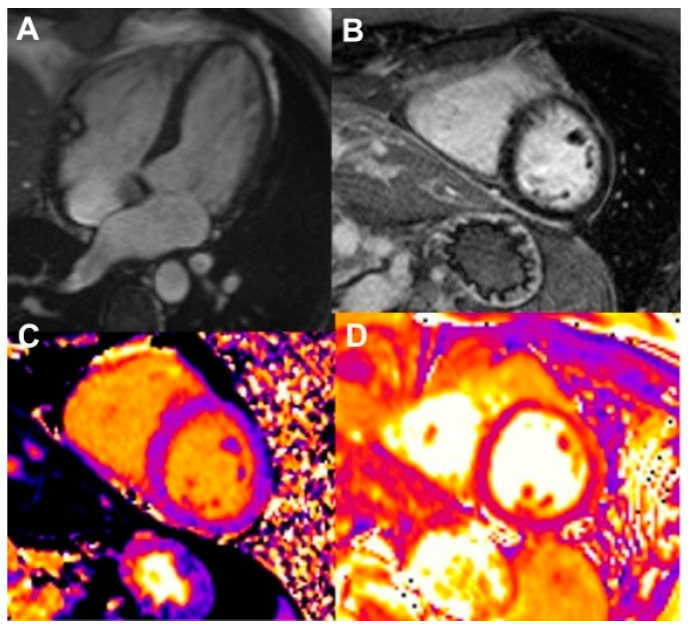
Immune checkpoint inhibitor (ICI)-associated myocarditis in a 58-year-old patient with melanoma treated with pembrolizumab. (**A**) Cine (bSSFP) four-chamber view which demonstrated mildly reduced left ventricular ejection fraction (49%). (**B**) Late gadolinium enhancement shows no evidence of myocardial scar. (**C**) Native T1 mapping in the four-chamber view reveals diffuse myocardial fibrosis, indicated by prolonged T1 relaxation time. (**D**) T2 mapping in the short-axis view demonstrates myocardial oedema, reflected by prolonged T2 relaxation time.

CMR represents a helpful additional imaging modality to distinguish between other potential etiologies of cardiomyopathy, such as myocarditis. Though ICI-related myocarditis is rare, its frequency is increasing with increased ICI usage. (Figure 6) It warrants close monitoring in at-risk patients and prompt investigation if suspected [88]. According to the recently updated Lake Louise Criteria, typical CMR features include myocardial oedema (prolonged T2) with a transmural midventricular to apical regional distribution pattern matching the areas of LV dysfunction, associated with nonischemic myocardial injury (based on T1 mapping, extracellular volume alterations and LGE) [89]. Supportive criteria are represented by pericardial effusion and systolic LV dysfunction [89]. However, there are limited data on its use in patients with ICI myocarditis. Most of these patients do not exhibit any significant LGE or myocardial oedema [90,91,92]. The largest study to date, a registry of 136 ICI myocarditis patients, showed that T1 mapping and the application of the modified Lake Louise Criteria provide important diagnostic value in this setting [46]. Abnormal T1 and T2 values were seen in 78% and 43% of the patients, respectively. A positive T1-based criterion was found in 95% of patients, so nearly all of the patients met the criteria for nonischemic myocardial injury using the modified Lake Louise Criteria [46]. Furthermore, native T1 values were independently associated with the subsequent development of major adverse cardiac events [46].

Therefore, together with cardiac biomarkers and electrocardiographic changes, CVMI will aid in the diagnostic algorithm of ICI-induced myocarditis and management.

##### Pericardial Disease

Acute pericarditis is a common side-effect of mediastinal RT, however it may also occur secondary to chemotherapy or imunothetapy. Moreover, patients with cancer may develop pericardial effusions due to the presence of metastatic disease in the pericardium. Multimodality CV imaging (echocardiography, CMR ± CT), ECG and measurement of cardiac biomarkers are recommended to confirm the diagnosis, assess the haemodynamic consequences of pericardial disease, and rule out associated myocarditis [1].

Despite its advantages, CMR has limitations, such as increased cost and lack of availability compared to echocardiography [70]. Moreover, CMR is contraindicated in patients with older cardiac metallic devices, and the results are less accurate in subjects with arrhythmias [72].

### 3.3. Nuclear Medicine Imaging

#### 3.3.1. Baseline Assessment

If TTE and CMR are unavailable for assessing LV EF or in patients with CMR-incompatible implanted devices, multi-gated acquisition nuclear imaging (MUGA) can be considered a third-line modality [1]. LV EF assessment using the MUGA method is highly reproducible, comparable to 3D echocardiography and CMR [93]. Although in the past MUGA has been the most common alternative to echocardiography in evaluating patients receiving chemotherapy, currently, its use in clinical practice is limited due to radiation exposure and the inability to obtain other important information (e.g., cardiac structures or GLS).

**Figure 7:**
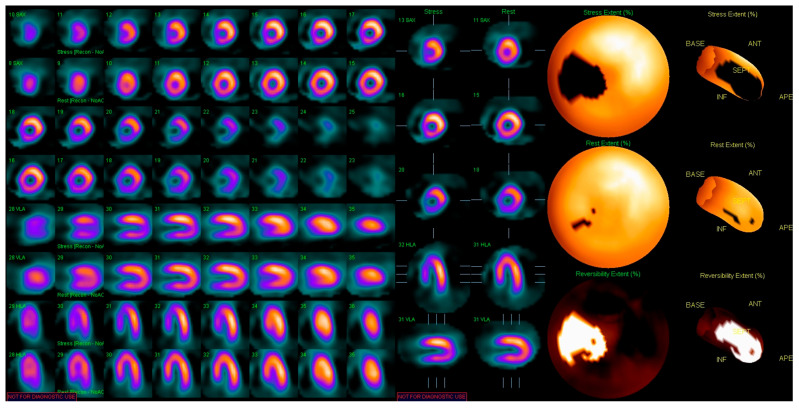
Myocardial perfusion SPECT imaging post-stress and at rest in a 76-year-old female patient with old anterior myocardial infarction and chronic LAD occlusion at coronarography: effort up to 95W, maximum HR of 145 bpm (99% of maximal HR), maximum BP 200/100 mmHg, no angina; resting BP 150/70 mmHg, resting HR 85 bpm. SPECT scan shows a reversible hypoperfusion area at the level of the interventricular septum, especially in the anterior territory, with a differential perfusion score of 11%. SPECT, single photon emission computed tomography; LAD, left anterior descending artery; HR, heart rate; BP, blood pressure.

Nuclear myocardial perfusion imaging should be performed to assess for myocardial ischaemia in symptomatic patients if clinical suspicion of CAD exists, especially before the use of cancer therapies associated with vascular toxicity. PET and SPECT are still the most frequently performed techniques for excluding or identifying the presence of stress-induced ischemia. (Figure 7) Myocardial perfusion imaging is useful not only for CAD diagnosis but also for the assessment of the hemodynamic significance of coronary stenosis, therapy guidance, and risk stratification in cancer patients [94].

#### 3.3.2. Identification of CTRCD

Nuclear medicine imaging is well-suited for identifying early myocardial dysfunction after cancer cardiotoxic treatments. The most significant advantage of nuclear medicine imaging is its high sensitivity, while one major limitation is the reduced spatial resolution.

Cardiac neuroimaging with Iodine-123 metaiodobenzylguanidine (123I-MIBG) can represent a scintigraphic image of the heart’s efferent sympathetic nervous innervations. A decrease in 123I-MIBG myocardial uptake has proved to be a strong predictor of cardiovascular death [95]. 123I-MIBG scintigraphy can be used to assess anthracycline-induced injury to myocardial adrenergic neurons during treatment [96]. Patients treated with anthracycline in a dose-dependent way showed an early reduction in 123I-MIBG uptake, which predicted late cardiotoxicity [97].

A specific anti-myosin antibody marked with 111In has been used to identify cardiomyocyte injury and necrosis in patients treated with anthracyclines, representing a predictor of LVEF decrease [98].

Although these techniques could be a tool for early detection of cardiotoxicity, their use remains limited to experimental settings.

#### 3.3.3. Amyloid Cardiomyopathy

**Figure 8:**
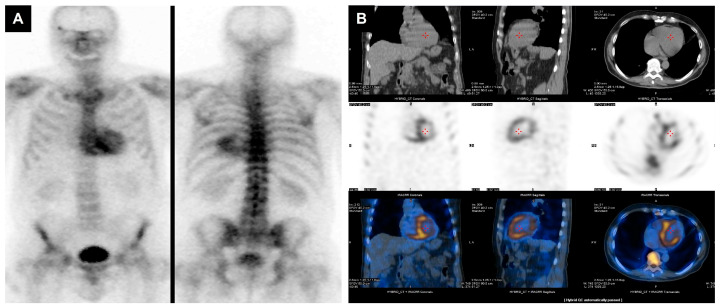
Planar and hybrid SPECT/CT bone scintigraphy in transthyretin cardiac amyloidosis. Anterior and posterior whole-body planar images (**A**) show intense myocardial tracer uptake exceeding rib activity, corresponding to Perugini grade 3 myocardial uptake, highly suggestive of transthyretin cardiac amyloidosis. Hybrid SPECT/CT images (**B**) in coronal, sagittal, and axial views confirm myocardial localisation of radiotracer signal, clearly distinct from blood pool or skeletal activity. The fused images (bottom row) demonstrate homogeneous radiotracer accumulation throughout the left ventricular myocardium, consistent with diffuse amyloid infiltration.

Infiltrative cardiomyopathy with light-chain amyloid may develop in patients with multiple myeloma or may progress from monoclonal gammopathy. Moreover, the prevalence of wild-type transthyretin cardiac amyloidosis is also higher than previously thought, especially in elderly patients in whom cancer prevalence is high [99]. In patients with suspected cardiac amyloidosis, echocardiography, CMR, and nuclear imaging complement the diagnostic workup. Imaging with 99mTc-pyrophosphate and 99 m^3^,3diphosphono-1,2-propanodicarboxylic acid helps identify transthyretin amyloidosis in most cases [100]. However, in the case of a positive nuclear scan, hematologic tests are mandatory, while cardiac or other tissue confirmation of the amyloid type might assist the final diagnosis, as nuclear imaging may also be positive in light-chain amyloidosis. (Figure 8)

#### 3.3.4. Inflammation

Inflammation is linked to both cancer and cardiovascular disease. With the increasing use of ICIs, myocardial inflammation will become an increasing problem. Studies have shown that 18F-fluorodeoxyglucose (18F-FDG) PET-CT scans play a significant role in diagnosing and staging cancer patients [101,102]. Research indicates that patients who have received doxorubicin and showed an increase in LV 18FDG uptake experienced a decline in LV EF [103]. This increased uptake suggests the presence of myocardial inflammation in CTRCD and merits further investigation despite this imaging technique’s high cost and limited availability. However, there are currently limited and inconsistent data on the use of FDG-PET in ICI myocarditis patients. Novel nuclear tracers are being investigated in this population. One such is Ga-DOTA(0)-Phe(1)-Tyr(3)-octreotide (Ga-DOTATOC) PET/CT, which utilises a somatostatin analogue to detect inflammation. This modality may help diagnose ICI myocarditis, as it can detect early inflammation that CMR may miss [104].

### 3.4. Cardiac Computed Tomography

#### 3.4.1. Baseline Assessment

The early recognition of increased atherosclerotic cardiovascular disease (ASCVD) risk is especially important. The need for a structured approach for ASCVD imaging in the cancer patient is critical to provide insight into strategies to reduce both short- and long-term CV events.

Cardiac computed tomography (CCT) can be helpful in both excluding and identifying subclinical cardiovascular disease, as well as assessing the severity of CAD in patients experiencing chest pain or LV dysfunction. Coronary angiography CCT with fractional flow reserve can evaluate the functional significance of coronary stenosis and congenital anomalous coronary arteries. Fractional flow reserve angio-CT for functional assessment of moderate stenoses can enhance risk stratification in cancer patients, and this strategy has also been shown to be cost-effective in the general population [105,106].

The coronary calcium score is an effective technique for risk stratification in asymptomatic cancer or survivor patients, particularly those with associated atherosclerotic cardiovascular risk factors. For patients with a low to intermediate pre-test probability of CAD, CCT serves as a reliable alternative modality with high sensitivity for ruling out obstructive CAD.

CT also plays a role in evaluating intracardiac masses during routine imaging performed for cancer staging, pericardial disease, and valvular function and disease. Moreover, it is useful when planning transcatheter valve intervention.

#### 3.4.2. Identification of CTRCD

Well established treatments such as radiotherapy (RT) can promote accelerated ASCVD [107]. Early detection and treatment of subclinical atherosclerosis in cancer survivors is recommended [1]. Standard serial CCT imaging provides an opportunity for surveillance of the evolution of ASCVD and can lead to decisions of early aggressive CV risk factor modification in prognostically favorable malignancies.

Moreover, CCT with myocardial late iodine enhancement can assess inflammation, fibrosis, and infiltrative processes in patients who are unable to undergo CMR.

In patients with cancer, exclusion of conditions that mimic acute coronary syndrome, such as 5-fluorouracil–associated vasospasm, ICI myocarditis, or takotsubo cardiomyopathy, may require anatomic or physiologic cardiac imaging. Coronary angio-CT is a particularly useful noninvasive method to exclude obstructive CAD in these particular acute coronary syndrome-like settings [108].

The main limitations of CCT are: radiation exposure; its use may be limited in more advanced renal disease that does not yet require dialysis due to iodinated contrast; those with contrast allergy require premedication; the presence of significant coronary calcifications may limit the assessment of coronary lesions severity; tachycardia and inability to perform breath-hold may limit image quality.

### 3.5. Role of CVMI in Assessing Late Cardiovascular Complications After Radiotherapy

Radiation exposure to the heart is common in patients treated for lymphoma and left breast, lung, and oesophagal cancer. Although proton beam has been introduced in the past decade, focusing more on the tumour and affecting less the surrounding structures, for many years, RT has been performed with photon radiation. RT exposure of the heart can be associated with CAD, valvular heart disease, pericarditis and pericardial effusion, restrictive cardiomyopathy, myocardial fibrosis, conduction abnormalities, and dysautonomia.

#### 3.5.1. Coronary Artery Disease

The risk and severity of CAD increase with radiation dose, larger volume exposed, younger age at the time of treatment, time from treatment, type of radiation source, and concurrent CV risk factors [109,110]. There is a linear radiation dose-to-risk of CAD relationship [110]. It is essential to monitor patients who received RT on the long term, as most complications occur more than 5 years after the therapy. For asymptomatic patients who received a mean heart dose of more than 15 Gy, non-invasive screening for CAD should be considered every 5 to 10 years, starting 5 years after radiation exposure [1].

Echocardiography provides a comprehensive evaluation of the cardiac structures and contractile function, with more relevant information being obtained from stress echocardiography. Other complementary modalities, such as CCT characterizing coronary anatomy, CMR, or perfusion nuclear imaging, can be applied.

#### 3.5.2. Valvular Heart Disease

RT can lead to significant valvular heart disease (VHD), resulting in thickening and fibrosis of the valvular apparatus [111]. The risk of developing valvular disease after RT is 34 times higher and typically manifests in the second decade following treatment [112]. In patients with Hodgkin lymphoma, a relationship between radiation dose to the heart and the development of valvular events after treatment has been described, especially at doses >30 Gy.

**Figure 9:**
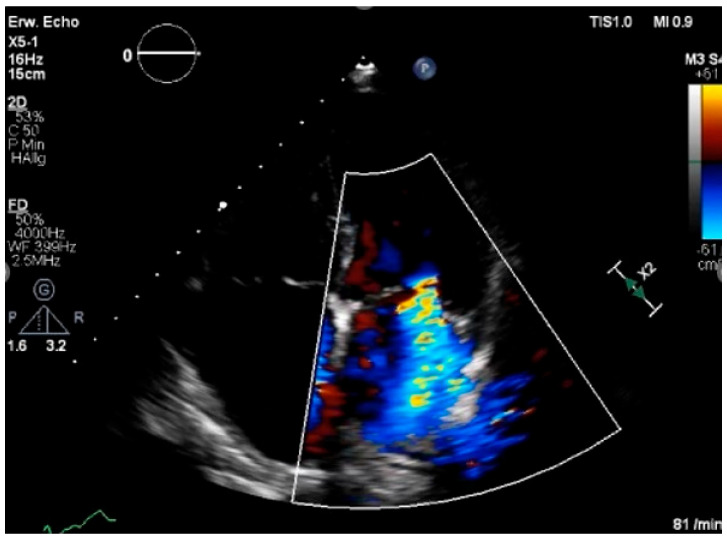
Valvular heart disease after chemotherapy. Case report: A 64-year-old female with NYHA class III–IV dyspnea was evaluated preoperatively in the cardio-oncology department following the simultaneous diagnosis of two tumours: a left-sided thoracic sarcoma and a right-sided breast cancer. Thoracic magnetic resonance imaging revealed a large mass in the left axilla (13.3 × 12.1 × 7.1 cm), suspected to involve the thoracic wall but without evidence of pulmonary infiltration. The patient had not received any prior medical or surgical treatment for the current malignancies at the time of evaluation. Her medical history was notable for a left-sided breast cancer 29 years earlier, treated with anthracyclines, radiation therapy, and breast-conserving surgery. Staging and histopathological data from the initial diagnosis were unavailable. Based on the tumour board’s recommendations, surgical resection of the sarcoma was planned, along with initiation of aromatase inhibitor therapy for the newly diagnosed breast cancer. During the cardio-oncology consultation, the patient’s overall cardiovascular risk, assessed using the HFA-ICOS score, was classified as very high. Transthoracic echocardiography revealed preserved left ventricular ejection fraction (LVEF 58%), grade III diastolic dysfunction, severe mitral regurgitation, and moderate aortic stenosis. Despite clinical recompensation, symptoms persisted, and mitral regurgitation remained severe (Videos S1–S4). Coronary angiography showed two-vessel coronary artery disease with a chronically occluded left circumflex artery, unsuitable for revascularisation. A diagnosis of heart failure with preserved ejection fraction (HFpEF) of mixed cardiotoxic and ischemic aetiology was made. Given the high surgical risk, an interdisciplinary heart team recommended transcatheter edge-to-edge mitral valve repair (TEER), which was successfully performed with minimal residual regurgitation (Videos S5–S7). Subsequently, the sarcoma was resected entirely, and hormone therapy for breast cancer was initiated. At the two-year follow-up, the patient reported new-onset dyspnea despite no evidence of cancer progression. Follow-up echocardiography showed a preserved LVEF of 55% but revealed newly developed severe aortic stenosis (Videos S8–S9). Repeat coronary angiography demonstrated progression of coronary artery disease with significant stenosis of the left anterior descending artery, requiring stent implantation. A renewed interdisciplinary evaluation recommended transcatheter aortic valve implantation (TAVI), which was successfully performed, resulting in a significant improvement in symptoms and resolution of dyspnea (Videos S10–S11). Conclusions: This case highlights the profound long-term impact of prior radiotherapy on cardiovascular toxicity in cardio-oncology patients. It underscores the role of previous radiotherapy not only in accelerating valvular and coronary artery disease, but also in predisposing to secondary malignancies. Importantly, it demonstrates that advanced transcatheter interventions, such as mitral TEER and TAVI, are feasible and effective treatment options for high-risk oncologic patients.

Echocardiography is the imaging method of choice for suspected valvular heart disease and for follow-up examinations after cancer therapy. (Figure 9, Videos S1–S11) RT-induced VHD exhibits a distinct pattern, characterized by fibrosis and calcification of the aortic root, aortic valve cusps, mitral valve annulus, and the base and mid portions of the mitral valve leaflets while sparing the mitral valve tips and commissures. Echocardiography may differentiate RT-induced VHD from rheumatic disease.

While two-dimensional TTE is sufficient to rule out relevant valvular diseases, transesophageal echocardiography utilizing three-dimensional acquisitions may be necessary to provide a more detailed description of the underlying pathology, especially in disorders of the mitral valve.

**Figure 10:**
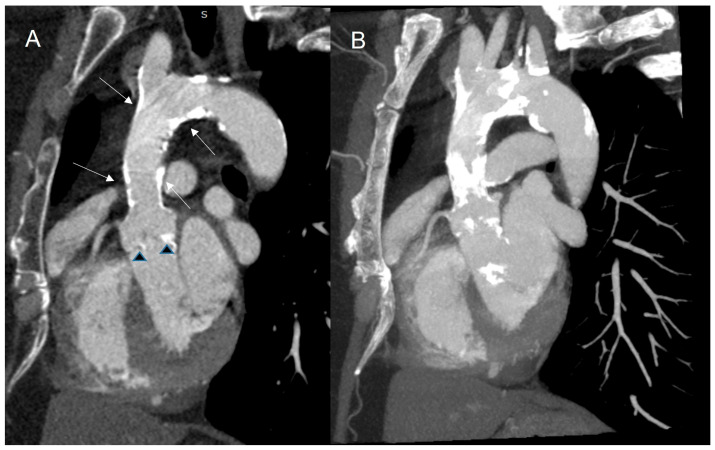
Calcifications of the ascending aorta and aortic valve after radiotherapy in a 46-year-old woman with Hodgkin’s lymphoma. (**A**) Oblique sagittal plane of the ascending aorta, transverse arch and proximal descending aorta. (**B**) Maximum intensity projection of the same plane. Extensive confluent calcifications of the ascending aorta and aortic arch—porcelain aorta (white arrows) as well as aortic valve calcification (arrowheads). CT, computer tomography.

The use of CT scans has become increasingly important, as they can evaluate aortic valve calcium score [113]. Additionally, patients who have undergone RT may experience mediastinal damage, including mediastinal fibrosis and a “porcelain aorta”, which can complicate an eventual cardiac surgery. (Figure 10).

Because of the high surgical risk of valvular interventions in RT patients, percutaneous techniques may be safer. CCT is well established as a tool in anatomic pre-planning for transcatheter-based valvular interventions, including aortic valve replacement.

#### 3.5.3. Pericardial Disease

Chronic pericarditis is a common side effect of mediastinal RT [1]. The incidence of this condition is related to the cumulative dose of radiation received. However, due to advancements in RT techniques and shielding methods, the incidence of radiation-induced pericarditis has significantly decreased [114]. In cases of chronic pericarditis, calcification of the pericardium may occur. CCT is the ideal imaging modality for assessing pericardial calcification and thickening due to its excellent spatial resolution [115].

## 4. Conclusions

Cardiotoxicity represents the most frequent cause of treatment interruption in cancer patients, with significant prognostic implications. Detecting patients at high risk for developing cardiotoxicity, as well as early diagnosis of CTRCD, have become essential in the management of cancer patients involving both cardiologists and oncologists. Multimodality cardiac imaging is essential for diagnosis and monitoring CTR-CVT.

LV EF monitoring remains the most used imaging technique to detect cardiotoxicity in clinical practice, but should be completed by STE derived GLS studies.

Even if still limited by costs and availability, CMR is a promising imaging technique, providing accurate functional and structural characterization even in patients with limited echogenicity.

CT has high value for coronary artery and pericardial evaluation, provided that radiation doses are sufficiently reduced.

Although nuclear imaging involves a higher radiation dose, it will remain essential for certain subsets of cancer patients, namely to monitor the baseline cancer stage and its progression. Incorporating additional cardiac-specific parameters, such as LV 18-FDG uptake, may help identify new CTRCD markers without causing inconvenience to patients.

## 5. Limitations and Future Directions

Much of imaging is still limited by operator and interpreter variability, as well as access.

Contrast-enhanced ultrasound molecular imaging could emerge as a novel imaging modality capable of tracking both temporal and spatial changes in tissues and blood vessels. This is crucial since anthracyclines can harm the myocardial vascular bed. However, this imaging modality is currently limited to research.

The use of artificial intelligence (AI) is a promising area that may improve accuracy and reproducibility and enhance efficiency across the spectrum of imaging modalities. Moreover, it may enable the integration of clinical and laboratory data with CVMI parameters to define individual phenotypes and better detect and predict the cardiotoxicity risk and potential treatment responses in oncological patients.

More data on genetic profiles for detecting early cardiovascular toxicity are warranted. Identifying genetic variations linked to cardiac damage caused by cancer-specific therapies could lead to more personalized and preventive strategies, enabling timely intervention and cardioprotective strategies.

## Figures and Tables

**Table 1:** The specific role of CVMI in several types of cardiotoxicity in cancer patients.

Modality Imaging	Myocardial Dysfunction	Myocarditis	Coronary Artery Disease	Pericardial Disease	Valvular Heart Disease
Echocardiography	First-line imaging test for 2D and Doppler cardiac structural and functional assessment 3D LV EF 2D/3D GLS Ultrasound enhancing agents to improve LV assessment when imaging is difficult	First-line imaging test for cardiac structural and functional assessment	First-line imaging test for cardiac function evaluation Stress echocardiography (exercise/dobutamine)—diagnosis and risk stratification	2D and Doppler TTE assessment	First-line imaging test—2D, 3D and Doppler TTE 2D and 3D TEE evaluation of cardiac valves
Strengths: - Easily accessible, low cost, lack of radiation, available from emergency room to the bedside Limitations: - Operator experience, LV EF interobserver variability - Strain assessments need to be performed on the same vendor machine during follow-up - Acoustic window may be difficult in certain patients (e.g., obesity, lung disease, mastectomy)					
CMR	LV EF assessment in patients with poor acoustic window/non-diagnostic TTE/no availability of 3D TTE T1 mapping -assessment for myocardial fibrosis and infiltrative processes such as cardiac amyloidosis T2 mapping -assessment for the presence of edema or inflammation LGE—inflammation, fibrosis, infiltrative process Strain techniques for subclinical cardiotoxicity	Gold standard for diagnosis of myocarditis related to antineoplastic therapies	Accurate assessment of cardiac function, including LV EF Strain techniques—evaluation for abnormal GLS causes Stress CMR—evaluation for CAD or microvascular disease, radiation-induced heart disease	Cine/steady-state free precession imaging Tissue-characterization of the pericardium	Cine/steady-state free precession imaging
Strengths: - higher reproducibility, superior image quality and resolution - cross-sectional imaging - ability to provide tissue-characterization of the LV and pericardium Limitations: - high cost, limited accessibility - severe renal impairment: gadolinium cannot be administered - less accurate results in subjects with arrhythmias or cardiac devices - contraindicated in patients with older cardiac devices					
Nuclear imaging	MUGA -assessment of LV EF Technetium pyrophosphate scan-diagnosis of cardiac transthyretin amyloidosis FDG PET/CT—may identify cardiac inflammation		Stress SPECT/PET—to exclude or identify the exercise/pharmacologic induced ischemia		
Strengths: - readily available with good access - MUGA measure of EF is highly reproducible - PET has superior accuracy for ischemia assessment in obese patients Limitations: - radiation - does not provide information about other cardiac structures					
CT	CCT with myocardial late iodine enhancement—can assess inflammation, fibrosis, and infiltrative processes in patients who are unable to undergo CMR		Coronary CTA -to exclude or identify CAD Coronary CTA—to assess CAD severity Coronary CTA—to evaluate CAD in ACS-like conditions (e.g., 5-FU, ICI, VEGFi or TKIs) Coronary CTA with FFR—evaluation of functional significance of coronary stenosis and congenital anomalous coronary CAC score -risk stratification for asymptomatic patients with cancer or survivors with CV risk factors	The ideal imaging modality for assessing pericardial calcification and thickening	Assessment of valvular function Useful for aortic valve calcium score Assessment of “porcelain aorta” Useful for transcatheter valve intervention pre-planning
Strengths: - Rapid and readily available - high negative predictive value in CAD evaluation - excellent spatial resolution - may identify and characterize high-risk plaque features Limitations: - radiation - high nefrotoxic risk of contrast agents in elderly, diabetic patients or those with significant renal impairment without dialysis - specific preparation protocols in patients with allergy to iodinated contrast agents - coronary lesions severity assessment may be limited by significant calcifications					

ACS, acute coronary syndromes; 2D, 2-dimensional; 3D, 3-dimensional; CAC, coronary artery calcium; CAD, coronary artery disease; CMR, cardiac magnetic resonance imaging; CT, computed tomography; CCT, cardiac computed tomography; CTA, computed tomographic angiography; EF, ejection fraction; FDG, fluorodeoxyglucose; FFR, fractional flow reserve; GLS, global longitudinal strain; LGE, late gadolinium enhancement; LV, left ventricular; MUGA, multigated acquisition scan; PET, positron emission tomography; RV, right ventricular; SPECT, single-photon emission computed tomography; TEE, transesophageal echocardiography; TTE, transthoracic echocardiography. All references to support each information in the present table are listed within the main narrative manuscript.

**Table 2:** Cancer therapy-related cardiac dysfunction definition (Modified after [1]).

**Symptomatic CTRCD**	Very severe	Advanced HF
	Severe	Recurrent HF hospitalizations
	Moderate	Need for outpatient HF therapy intensification
	Mild	Mild HF symptoms
**Asymptomatic CTRCD**	Severe	New LVEF reduction to < 40%
	Moderate	New LVEF reduction by ≥10 percentage to an LVEF of 40–49% OR New LVEF reduction by <10 percentage to an LVEF of 40–49% AND either new relative decline in GLS by >15% from baseline OR new rise in cardiac biomarkers
	Mild	LVEF ≥ 50% AND new relative decline in GLS by >15% from baseline AND/OR new increase in cardiac biomarkers

CTRCD, cancer therapy-related cardiac dysfunction; HF, heart failure; LVEF, left ventricular ejection fraction; GLS, global longitudinal strain.

**Table 3:** Anthracycline chemotherapy TTE surveillance protocol (adapted after [1]).

	**Baseline**	**C1**	**C2**	**C3**	**C4**	**C5**	**C6**	**3M Post tx**	**12M Post tx**
Low risk	+++				+				+++
Moderate risk	+++				++				+++
High and very high risk	+++		+++		+++		+++	+++	+++

TTE, transthoracic echocardiography; C, chemotherapy cycle; M, months; tx, treatment. Class of recommendation: +++, Class I; ++, Class IIa; +, Class IIb.

**Table 4:** HER2-targeted therapy TTE surveillance protocol (adapted after [1] 1 ESC).

	Baseline	3M	6M	9M	12M	3M Post tx	12m Post tx
Low and moderate risk	+++	+++	+++	+++	+++		+++
High and very high risk	+++	+++	+++	+++	+++	+++	+++

TTE, transthoracic echocardiography; M, months; tx, treatment; Class of recommendation: +++, Class I.

**Table 5:** VEGFi TTE surveillance protocol (adapted after [1]).

	Baseline	3M	4M	6M	8M	9M	12M Post tx	Every 6–12M
Low risk	++							
Moderate risk	++		+		+		+	++
High and very high risk	+++	++		++		++	++	++

TTE, transthoracic echocardiography; M, months; tx, treatment; Class of recommendation: +++, Class I; ++, Class IIa; +, Class IIb.

**Table 6:** Immune checkpoint inhibitors TTE surveillance protocol (adapted after [1]).

	Baseline	C2	C3	C4	Every 3C ^a^	Every 6M–12M ^b^
Low risk	+					
High risk	+++					

TTE, transthoracic echocardiography; C, chemotherapy cycle; M, months; Class of recommendation: +++, Class I; +, Class IIb. ^a^ Every three cycles until completion of therapy to detect subclinical ICI-related cardiovascular toxicity. ^b^ In patients who require long-term (>12 months) ICI treatment.

## Data Availability

No new data were created or analyzed in this study. Data sharing is not applicable to this article.

## Supplementary Materials

The following supporting information can be downloaded at: https://www.mdpi.com/article/10.3390/jcdd13010027/s1, Video S1: Transthoracic echocardiographic cine loop in the parasternal long-axis view with colour Doppler demonstrating calcification of the mitral and aortic valves, with severe mitral regurgitation; Video S2: Transthoracic echocardiographic cine loop in the apical four-chamber view with colour Doppler demonstrating severe primary mitral regurgitation involving the mitral valve; Video S3: Transoesophageal echocardiographic cine loop in the mid-oesophageal bicommissural view at 65° demonstrating severe primary mitral regurgitation; Video S4: Transoesophageal echocardiographic cine loop in the mid-oesophageal view at 45° demonstrating calcification of the aortic cusps secondary to radiotherapy, with moderate aortic valve stenosis; Video S5: Transoesophageal echocardiographic cine loop in the mid-oesophageal bicommissural view at 55° demonstrating intraprocedural fixation of the device for edge-to-edge repair of severe primary mitral regurgitation; Video S6: Fluoroscopic intraprocedural view demonstrating the edge-to-edge repair device positioned at the mitral valve; Video S7: Transthoracic echocardiographic cine loop in the apical four-chamber view with colour Doppler demonstrating mild residual mitral regurgitation following edge-to-edge repair; Video S8: Transthoracic echocardiographic cine loop in the parasternal long-axis view demonstrating progressive calcification of the aortic valve and a satisfactory result following edge-to-edge repair of the mitral valve; Video S9: Transoesophageal echocardiographic cine loop in the mid-oesophageal view at 40° with focus on the aortic valve, demonstrating calcification of the aortic cusps secondary to radiotherapy and severe aortic valve stenosis; Video S10: Transthoracic echocardiographic cine loop in the apical three-chamber view demonstrating an excellent result following edge-to-edge repair of the mitral valve and transcatheter aortic valve replacement; Video S11: Transthoracic echocardiographic cine loop in the apical three-chamber view with colour Doppler demonstrating an excellent result following edge-to-edge repair of the mitral valve and transcatheter aortic valve replacement.
